# Late-onset multiple acyl-CoA dehydrogenase deficiency mimicking myositis in an elderly patient: a case report

**DOI:** 10.1186/s12883-020-02010-w

**Published:** 2020-12-02

**Authors:** Yiming Zheng, Yawen Zhao, Wei Zhang, Zhaoxia Wang, Yun Yuan

**Affiliations:** Neurology Department, Peking University First Hospital, No. 8 Xishiku Street, Xicheng District, Beijing, 100034 China

**Keywords:** MADD, Myositis, Lipid storage myopathy, Case report

## Abstract

**Background:**

Late-onset multiple acyl-CoA dehydrogenase deficiency (MADD) is a rare and treatable inherited lipid storage myopathy. Here, we report an elderly patient with MADD mimicking myositis.

**Case presentation:**

An 80-year-old woman had progressive weakness in her limbs, exercise intolerance, and no muscle pain for 3 months. The patient’s serum creatine kinase level was slightly elevated. The initial diagnosis was myositis. However, muscle biopsy showed many cytoplasmic vacuoles stained with oil red O, indicating the presence of lipid storage myopathy. The plasma acylcarnitine profile showed increased medium-chain and long-chain acylcarnitine species, consistent with the diagnosis of MADD. Riboflavin treatment dramatically improved muscle weakness.

**Conclusions:**

MADD should be considered when evaluating elderly patients with subacute muscle weakness.

**Supplementary Information:**

The online version contains supplementary material available at 10.1186/s12883-020-02010-w.

## Background

Late-onset multiple acyl-CoA dehydrogenase deficiency (MADD) is a rare heterogeneous inherited disease of fatty acid oxidation that can cause neuromuscular symptoms in adults and responds well to riboflavin treatment [1]. MADD is difficult to diagnose in elderly patients and may be missed without a muscle biopsy [2]. Here, we describe a case of elderly-onset MADD mimicking myositis, highlighting the need to be alert for hereditary myopathy, especially MADD, as a differential diagnosis of myositis in elderly patients.

## Case presentation

An 80-year-old Chinese woman presented with progressive weakness in the proximal limbs and exercise intolerance for 3 months. She had difficulties in climbing stairs and getting up from a chair and needed to rest after walking about 100 m. Her symptoms continued to worsen. Two months before admission, it was difficult for the patient to raise her arms to collect objects or comb her hair. She could only walk about 10 m unassisted. She also complained of numbness in her toes and fingers. She had no ptosis and no difficulty chewing or swallowing. She exhibited no skin rashes, muscle pain, or weakness fluctuations. The patient had lost about 10 kg of weight in the past year. She had a 10-year history of hypertension. She used sertraline to treat anxiety for about 3 years. She had no history of myopathy or taking statins. Her family history was unremarkable. Neurological evaluation showed a waddling gait and muscle weakness in the patient’s neck flexors and bilateral proximal limbs (4/5 by Medical Research Council scale). No sensory abnormality was found. Serum creatine kinase was slightly elevated at 361 U/L (reference range 25–170 U/L), and lactate dehydrogenase was also elevated at 500 U/L (100-240 U/L). Myositis-specific antibody and associated antibody tests showed that the anti-signal recognition particle antibody and anti-Ku antibody were weakly positive (Supplementary Figure S1). A nerve conduction study showed that the conduction velocity of bilateral median and superficial peroneal nerves was decreased. The electromyography and repetitive nerve stimulation were normal. Computed tomography of the chest and abdomen showed no signs of tumors. Magnetic resonance imaging of thigh muscles revealed mild fatty infiltration and edema changes (Supplementary Figure S2).

**Fig. 1:**
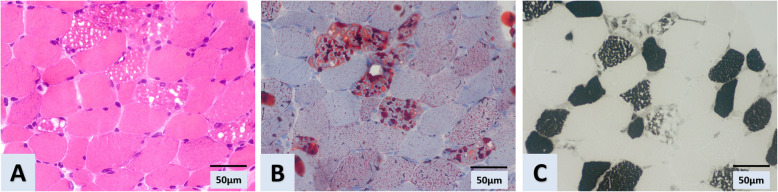
Muscle biopsy of the vastus lateralis muscle indicating lipid storage myopathy. Light microscopy of muscle biopsy showing muscle fibers containing cytoplasmic vacuoles under hematoxylin and eosin staining (**a**). Oil red O staining of vacuoles reveals lipid accumulation (**b**), which mainly appears in type 1 muscle fibers when stained with ATPase pH 4.4 (**c**)

The initial diagnosis was myositis, and a muscle biopsy was performed to confirm the diagnosis and rule out other myopathies. However, a biopsy of the vastus lateralis muscle showed many cytoplasmic vacuoles under hematoxylin and eosin staining that were confirmed as lipid droplets when stained with oil red O. These mainly appeared in type 1 muscle fibers when stained with ATPase pH 4.4, indicating lipid storage myopathy without any features of myositis (Fig. 1). The plasma acylcarnitine profile under the fed state showed increased medium-chain and long-chain acylcarnitine species (decanoyl (C10) carnitines, 0.54umol/L (normal range: 0–0.50umol/L); tetradecadienoyl (C14) carnitine, 0.09umol/L (0–0.04umol/L) and hexadecenoyl (C16) carnitine, 0.48umol/L (0.02–0.42umol/L)), consistent with the diagnosis of MADD. The patient received oral vitamin B2 (riboflavin) at a dose of 20 mg three times a day. Her muscle weakness gradually improved. After a month of treatment, she regained her normal muscle strength. The patient refused a further genetic test to confirm the genotype.

## Discussion and conclusions

To the best of our knowledge, this report describes the oldest patient ever diagnosed with MADD. The patient had not experienced muscular symptoms previously and began to manifest muscle weakness only in her eighties.

MADD is easily misdiagnosed as myositis [3]. Taking into account the patient’s age and subacute onset, progressive weakness of the proximal limbs, elevated creatine kinase, positive anti-signal recognition particle antibody, and edema changes indicated by magnetic resonance imaging of the thigh muscle, the initial diagnosis was myositis, including immune-mediated necrotizing myopathy or sporadic inclusion body myositis. However, exercise intolerance and peripheral nerve damage could not be explained by a diagnosis of myositis. As observed in our patient, MADD can also include distal sensory neuropathy [4]. Early diagnosis is crucial because MADD can be treated with riboflavin supplementation, which may induce a dramatic response [5].

In conclusion, MADD should be included in the differential diagnosis of subacute-onset proximal muscle weakness even in elderly patients, especially those with extreme fatigue. Plasma acylcarnitine profile and urinary organic acid, or muscle biopsy indicating lipid storage myopathy, are important factors to evaluate to avoid overlooking a diagnosis of MADD.

## Supplementary Information

**Additional file 1.**

## Data Availability

All data related to this case report are documented within this manuscript.

## Abbreviation

MADD: Multiple acyl-CoA dehydrogenase deficiency
