# Supplementary material for: Late-onset multiple acyl-CoA dehydrogenase deficiency mimicking myositis in an elderly patient: a case report
Source: BMC Neurol. 2020 Dec 2;20:436. doi: 10.1186/s12883-020-02010-w (PMC7709274; doi:10.1186/s12883-020-02010-w)
Supplement: Supplementary file 1 — Additional file 1. [file 12883_2020_2010_MOESM1_ESM.docx]

**Supplementary Figure S1**: The myositis-specific autoantibodies and myositis-associated autoantibodies profile
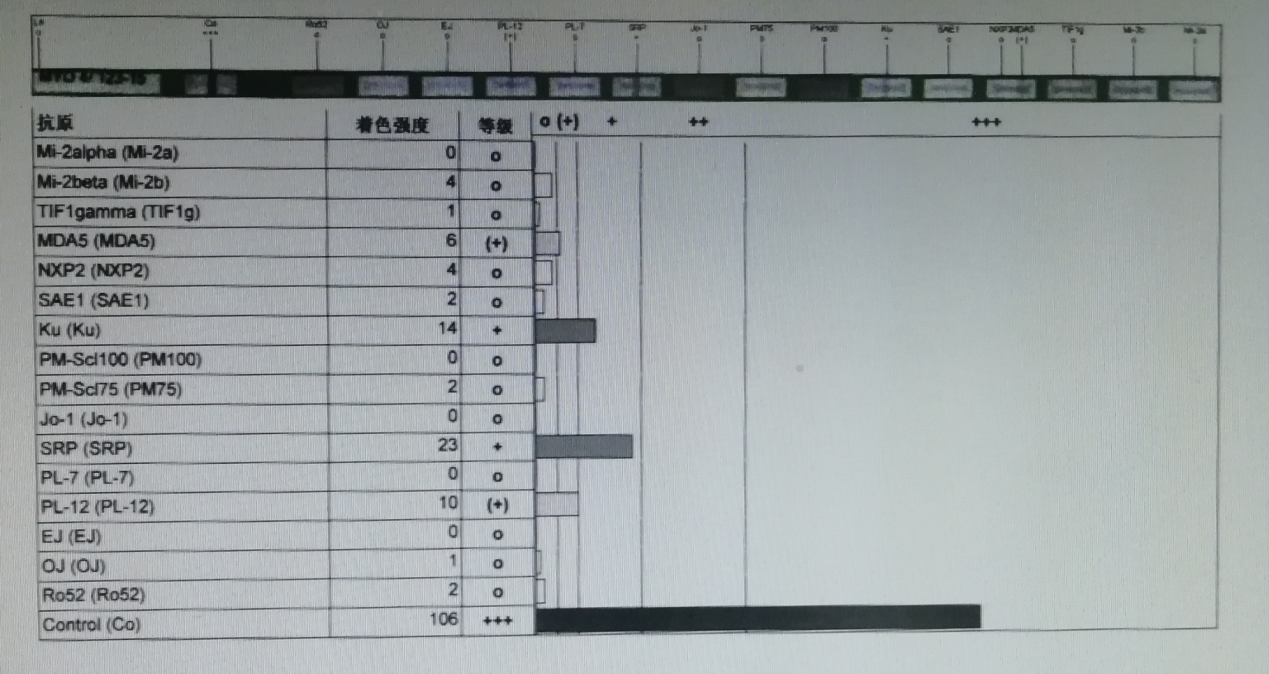


Legend: The myositis-specific autoantibodies and myositis-associated autoantibodies detected using commercial line immunoblot assay showed that anti-SRP antibody and anti-Ku antibody were weakly positive (+) .

**Supplementary Figure S2**: The thigh muscle MRI of the patient


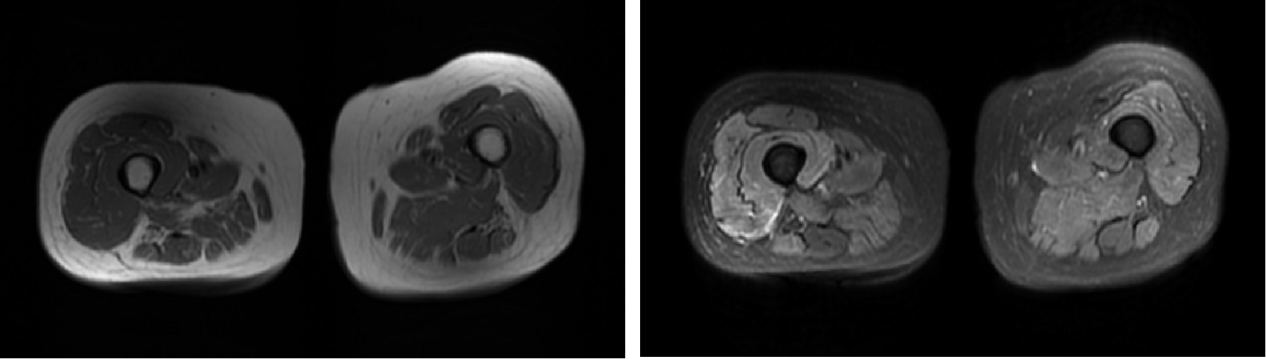


Legend: The thigh muscle MRI showing mild fatty infiltration (left, T1 weighted sequence) and edema changes (right, STIR sequence) in this patient.
